# P-1625. SARS-CoV-2 Viral Load Dynamics in Participants With Solid Organ Transplantation and Severely Reduced Kidney Function From the Remdesivir Phase 3 REDPINE Study Who Were Hospitalized for COVID-19

**DOI:** 10.1093/ofid/ofaf695.1801

**Published:** 2026-01-11

**Authors:** Lauren Rodriguez, Shuguang Chen, Yu Hu, Jasmine Moshiri, Ross Martin, Yiannis Koullias, Charlotte Hedskog

**Affiliations:** Gilead Sciences, Inc., Foster City, CA; Gilead Sciences, Inc, Foster City, California; Gilead Sciences, Inc., Foster City, CA; Gilead Sciences, Inc., Foster City, CA; Gilead Sciences, Foster City, California; Gilead Sciences, Inc., Foster City, CA; Gilead Sciences, Inc., Foster City, CA

## Abstract

**Background:**

Prolonged shedding of SARS-CoV-2 has been documented in viral dynamics studies of immunocompromised patients. Remdesivir (RDV) is a nucleotide analog prodrug approved to treat COVID-19 in hospitalized and nonhospitalized patients. Here we present SARS-CoV-2 viral load (VL) analyses in participants with solid organ transplantation (SOT) from the Phase 3 REDPINE study.

**Methods:**

REDPINE was a double-blind, placebo-controlled trial in participants hospitalized for COVID-19 with severely reduced kidney function who were randomized 2:1 to receive RDV or placebo (PBO) for 5 days. Out of 243 participants enrolled and treated in the study, forty-two participants had SOT and received immunosuppressive drugs prior to and during the study. Nasopharyngeal swab samples were collected on days 1 (baseline), 3, 5, 7, 14, 21, and/or 29. The change from baseline in SARS-CoV-2 RNA VL was compared in an ad hoc analysis between the RDV and PBO groups and between participants with and without SOT in the RDV group using a mixed model repeated measures (MMRM) approach.

**Results:**

Overall, RDV treatment led to significantly greater reductions from baseline in VL on Day 5 (least-squares mean [LSM] difference, −0.48 log_10_ copies/mL; *P* = 0.0332) and Day 7 (−0.57 log_10_ copies/mL; *P* = 0.0335) compared to PBO. In the RDV-treated group, changes from baseline in VL (log_10_ copies/mL) for participants with and without SOT, respectively, were as follows: Day 5, –0.69 and –1.45; Day 7, –0.85 and –2.02; Day 14, –1.06 and –2.40. Participants with SOT showed slower VL reductions from baseline on Days 5, 7, and 14 compared to participants without SOT (*P* ≤0.0191). In RDV-treated participants without SOT, 14/20 (70%) reached the lower limit of quantification (LLOQ) of the RT-qPCR VL assay (3.35 log_10_ copies/mL) at Day 14, whereas 3/11 (27%) participants with SOT reached the LLOQ at Day 14.

**Conclusion:**

In the REDPINE study, RDV treatment resulted in reduced SARS-CoV-2 VL compared to PBO. In RDV-treated participants with SOT, VL reductions were slower compared to participants without SOT, potentially due to diminished immune system support to clear the virus. These findings suggest that extending the duration of RDV treatment beyond 5 days may be beneficial in this patient population to mitigate prolonged viral shedding.

**Disclosures:**

Lauren Rodriguez, PhD, Gilead Sciences, Inc.: Employee|Gilead Sciences, Inc.: Stocks/Bonds (Public Company) Shuguang Chen, PhD, Gilead Sciences, Inc.: Employee|Gilead Sciences, Inc.: Stocks/Bonds (Public Company) Yu Hu, PhD, Gilead Sciences, Inc.: Employee|Gilead Sciences, Inc.: Stocks/Bonds (Public Company) Jasmine Moshiri, PhD, Gilead Sciences, Inc.: Employee|Gilead Sciences, Inc.: Stocks/Bonds (Public Company) Ross Martin, PhD, Gilead Sciences, Inc.: Employee|Gilead Sciences, Inc.: Stocks/Bonds (Public Company) Yiannis Koullias, MD, Gilead Sciences, Inc.: Employee|Gilead Sciences, Inc.: Stocks/Bonds (Public Company) Charlotte Hedskog, PhD, Gilead Sciences, Inc.: Employee|Gilead Sciences, Inc.: Stocks/Bonds (Public Company)

